# Metabolic selection of a homologous recombination-mediated gene loss protects *Trypanosoma brucei* from ROS production by glycosomal fumarate reductase

**DOI:** 10.1016/j.jbc.2021.100548

**Published:** 2021-03-17

**Authors:** Marion Wargnies, Nicolas Plazolles, Robin Schenk, Oriana Villafraz, Jean-William Dupuy, Marc Biran, Sabine Bachmaier, Hélène Baudouin, Christine Clayton, Michael Boshart, Frédéric Bringaud

**Affiliations:** 1 Univ. Bordeaux, CNRS, Microbiologie Fondamentale et Pathogénicité (MFP), UMR 5234, Bordeaux, France; 2 Univ. Bordeaux, CNRS, Centre de Résonance Magnétique des Systèmes Biologiques (CRMSB), UMR 5536, Bordeaux, France; 3 Fakultät für Biologie, Genetik, Ludwig-Maximilians-Universität München, Martinsried, Germany; 4 Univ. Bordeaux, Plateforme Protéome, Bordeaux, France; 5 Zentrum für Molekulare Biologie der Universität Heidelberg (ZBMH), Universität Heidelberg, Heidelberg, Germany

**Keywords:** *trypanosoma*, genomic rearrangement, homologous recombination, NADH-dependent fumarate reductase (FRD), phosphoenolpyruvate carboxykinase (PEPCK), covalent flavinylation, Cytb5R domain, reactive oxygen species, positive selection, parasite differentiation, ACN, acetonitrile, FRD, fumarate reductase, PCF, procyclic form, PEP, phosphoenolpyruvate, PEPCK, phosphoenolpyruvate carboxykinase, PPDK, pyruvate phosphate dikinase, ROS, reactive oxygen species

## Abstract

The genome of trypanosomatids rearranges by using repeated sequences as platforms for amplification or deletion of genomic segments. These stochastic recombination events have a direct impact on gene dosage and foster the selection of adaptive traits in response to environmental pressure. We provide here such an example by showing that the phosphoenolpyruvate carboxykinase (*PEPCK*) gene knockout (Δ*pepck*) leads to the selection of a deletion event between two tandemly arranged fumarate reductase (*FRDg* and *FRDm2*) genes to produce a chimeric *FRDg-m2* gene in the Δ*pepck∗* cell line. FRDg is expressed in peroxisome-related organelles, named glycosomes, expression of FRDm2 has not been detected to date, and FRDg-m2 is nonfunctional and cytosolic. Re-expression of FRDg significantly impaired growth of the Δ*pepck∗* cells, but FRD enzyme activity was not required for this negative effect. Instead, glycosomal localization as well as the covalent flavinylation motif of FRD is required to confer growth retardation and intracellular accumulation of reactive oxygen species (ROS). The data suggest that FRDg, similar to *Escherichia coli* FRD, can generate ROS in a flavin-dependent process by transfer of electrons from NADH to molecular oxygen instead of fumarate when the latter is unavailable, as in the Δ*pepck* background. Hence, growth retardation is interpreted as a consequence of increased production of ROS, and rearrangement of the *FRD* locus liberates Δ*pepck∗* cells from this obstacle. Interestingly, intracellular production of ROS has been shown to be required to complete the parasitic cycle in the insect vector, suggesting that FRDg may play a role in this process.

Trypanosomatids, including the human infective *Leishmania* and *Trypanosoma* species, present several biological singularities in comparison with classical eukaryotic model organisms. For instance, genes are transcribed constitutively as part of long polycistronic units where the precursor mRNA molecules are matured by coupled trans-splicing and polyadenylation (1). As a consequence, gene regulation occurs mostly at the posttranscriptional, translational, and posttranslational levels with no control at the level of transcription initiation. Changes in gene copy number can also modulate gene expression and are therefore seen when selective pressure is applied. They usually arise from homologous recombination events between repeated sequences and are particularly common in *Leishmania* spp (2). In *Leishmania*, small repetitive sequences are widespread throughout the genome and recombination events appear stochastically with a frequency in the order of 10^−6^/10^−7^ per cell generation. They result either in the production of extrachromosomal DNA sequences or in the deletion of the DNA fragment located between the two recombinogenic repeats. Under selection pressure, such as exposition to drugs, a subpopulation with an advantageous amplicon conferring drug resistance can emerge (3, 4, 5, 6, 7, 8, 9). The genome of *Trypanosoma brucei* also contains a large number of sequence repeats (773) potentially leading to 1848 genetic recombination events, some of them already experimentally validated (2). So far, no DNA amplification (except for changes in ploidy and in gene copy number) has been observed upon specific selection, suggesting that deletions are more common (10, 11, 12). We report here the selection of such a stochastic deletion in the genome of *T. brucei* mutants, which is driven by metabolic constraints.

The procyclic form (PCF) of *T. brucei* has an elaborate energy metabolism based on glucose or proline, depending on carbon source availability (13). In the glucose-free environment of its insect host (tsetse fly), the parasite depends on proline for its metabolism (14, 15) and needs to produce hexose phosphates through gluconeogenesis from proline-derived phosphoenolpyruvate (PEP) to feed essential pathways (16). Two phosphoenolpyruvate-producing enzymes, PEP carboxykinase (PEPCK, EC: 4.1.1.32, Tb927.2.4210) and pyruvate phosphate dikinase (PPDK, EC 2.7.9.1, Tb927.11.3120) have a redundant function for the essential gluconeogenesis from proline (17). In glucose-rich conditions, PPDK and PEPCK work in the opposite direction to produce pyruvate and oxaloacetate, respectively, in addition to ATP. This pathway is also essential to maintain the glycosomal redox balance (18). Glycosomes are peroxisome-related organelles using the same machinery for protein import and harbor the 6 or 7 first glycolytic steps (19). Because of the impermeability of the glycosomal membrane to bulky metabolites, such as cofactors and nucleotides, ATP molecules consumed by the first glycolytic steps (steps 1 and 3 in Fig. 1) need to be regenerated in the glycosomes by PPDK and PEPCK (step 14 and 15) (18). Similarly, NAD^+^ molecules consumed in the glycosomes during glycolysis (step 6) have to be regenerated within the organelle by the succinic fermentation pathway composed of PEPCK, malate dehydrogenase (EC: 1.1.1.37, Tb927.10.15410, step 16), fumarase (EC: 4.2.1.2, Tb927.10.15410, step 17), and NADH-dependent fumarate reductase (FRDg, EC: 1.3.1.6, Tb927.5.930, step 18) (20). Alternatively, the glycosomal redox balance can be maintained by the glycerol 3-phosphate (Gly3P)/dihydroxyacetone phosphate (DHAP) shuttle, as observed for the PEPCK null mutant (Δ*pepck*) and illustrated in Figure 1*B* (20).

**Figure 1:**
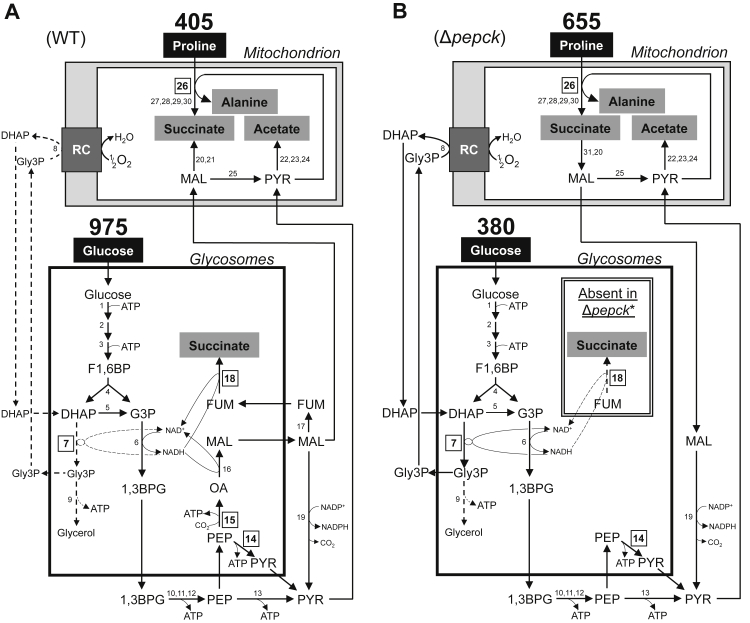
**Schematic representation of the central metabolism of the wild-type and Δ*pepck* mutant PCF cell lines in glucose-rich medium**. This figure highlights the catalytic steps from glucose and proline metabolism in the wild-type (WT) (*A*), Δ*pepck* and Δ*pepck∗* (*B*) cell lines. The mitochondrial pathways have been considerably simplified, in particular the respiratory chain (RC) represented by a *gray box*. Excreted end products from degradation of glucose and proline are in a *gray* background and *dashed lines* represent enzymatic steps not used or used at a background noise level. *Boxed* numbers correspond to the enzymatic steps investigated. The rates of glucose and proline consumption (nmol h^−1^ mg^−1^ of protein) indicated above the carbon source names are deduced from Tables S1 and are consistent with previous data (18). The *double box* inside the glycosomes including the FRDg step is missing in the Δ*pepck*∗ cell line. Enzymes: 1, hexokinase; 2, glucose-6-phosphate isomerase; 3, phosphofructokinase; 4, aldolase; 5, triose-phosphate isomerase; 6, glyceraldehyde-3-phosphate dehydrogenase; 7, glycosomal NADH-dependent glycerol-3-phosphate dehydrogenase (GPDH); 8, mitochondrial FAD-dependent glycerol-3-phosphate dehydrogenase (GPDH); 9, glycerol kinase; 10, phosphoglycerate kinase; 11, phosphoglycerate mutase; 12, enolase; 13, pyruvate kinase; 14, pyruvate phosphate dikinase (PPDK); 15, phosphoenolpyruvate carboxykinase (PEPCK); 16, glycosomal malate dehydrogenase; 17, fumarase; 18, glycosomal NADH-dependent fumarate reductase (FRDg); 19, cytosolic malic enzyme; 20, mitochondrial fumarase; 21, mitochondrial NADH-dependent fumarate reductase (FRDm1); 22, pyruvate dehydrogenase complex; 23, acetate:succinate CoA-transferase; 24, acetyl-CoA thioesterase; 25, mitochondrial malic enzyme; 26, proline dehydrogenase (PRODH); 27, pyrroline-5 carboxylate dehydrogenase; 28, alanine aminotransferase; 29, α-ketoglutarate dehydrogenase complex; 30, succinyl-CoA synthetase; 31, succinate dehydrogenase. 1,3BPG, 1,3-biphosphoglycerate; DHAP, dihydroxyacetone phosphate; F1,6BP, fructose 1,6-bisphosphate; FUM, fumarate; G3P, glyceraldehyde 3-phosphate; Gly3P, glycerol 3-phosphate; MAL, malate; OA, oxaloacetate; PEP, phosphoenolpyruvate; PYR, pyruvate; RC, respiratory chain.

The *T. brucei* genome contains three *FRD* genes. Two are tandemly arranged in chromosome 5: they encode the glycosomal isoform (FRDg) and a potential FRD isoform for which expression has not been detected so far in trypanosomes (FRDm2, Tb927.5.940). The third gene, located on chromosome 10, codes for the mitochondrial isoform (FRDm1, Tb927.10.3650, step 21) (21, 22) (Fig. 2*B*). FRDg is a 120 kDa protein composed of three domains, a N-terminal ApbE (Alternative pyrimidine biosynthesis protein)-like flavin transferase domain (pfam: PF02424), a central FRD domain (superfamily: SSF56425), and a C-terminal cytochrome b5 reductase (Cytb5R) domain (superfamily: SSF63380) (21). In addition, FRDg has a conserved flavinylation motif at its extreme N terminus, shown to be required for FRD activity in the related organism *Leptomonas pyrrhocoris* (23). We report here that two independent PEPCK null mutant cell lines express a chimeric nonfunctional FRDg-m2 isoform resulting from homologous recombination within the *FRDg*/*FRDm2* locus. The selective advantage provided by the loss of the *FRDg* gene in the context of the PEPCK null background depends on the glycosomal localization of FRDg and the presence of the N-terminal putative flavinylation site. We propose that the absence of metabolic flux through the glycosomal succinic fermentation pathway in PEPCK null mutants made the FAD/FMN cofactors of FRDg available to oxygen for production of reactive oxygen species (ROS) in the organelles.

**Figure 2:**
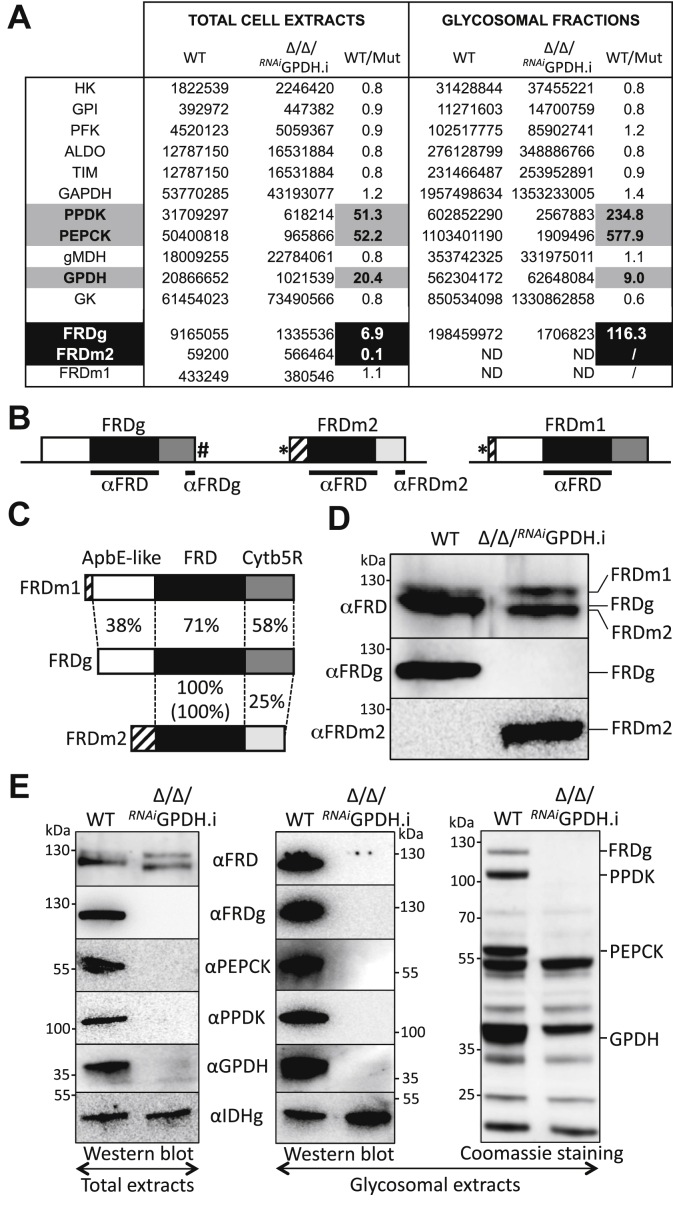
**Altered expression of the FRD isoforms in the Δ*ppdk*/Δ*pepck*/**^***RNAi***^**GPDH mutant cell line.***A*, compares the expression of glycosomal glycolytic enzymes and FRD isoforms obtained by label-free mass spectrometry proteomic analysis (n = 3) of total lysates and glycosomal fractions of the parental (WT) and tetracycline-induced Δ*ppdk*/Δ*pepck*/^*RNAi*^GPDH (Δ/Δ/^*RNAi*^GPDH.i) cell lines (see the PXD020185 data set in the PRIDE partner repository). The ratio between peptide counts in the parental and mutant cell lines is indicated in the WT/Mut column, with those showing big differences being highlighted. The organization of *FRD* genes in the *T. brucei* genome is shown in (*B*) with the glycosomal FRDg and the putative mitochondrial FRDm2 isoforms tandemly arranged on chromosome 5, while the mitochondrial FRDm1 isoform is located on chromosome 10. The *white*, *black*, and *gray* (*light* and *dark*) *boxes* represent the ApbE-like, fumarate reductase, and cytochrome b5 reductase domains, respectively. Mitochondrial targeting signals present at the N-terminus extremity of FRDm1 (experimentally confirmed (22)) and FRDm2 (putative signal corresponding to most of the hatched *box*) are indicated by asterisks, and the PTS1 motif at the C-terminal end of FRDg is highlighted by a hash. The recombinant protein (αFRD) and peptides (αFRDg and αFRDm2) used for immune sera production are indicated by *black bars*. *C*, indicates amino acid identity between the three domains shared by the FRD isoforms expressed as percentage, with the value into brackets corresponding to nucleotide identity. Expression of the FRD isoforms in the parental (WT) and Δ/Δ/^*RNAi*^GPDH.i cell lines is shown in (*D*) by *western blot*, using antibodies specific for FRDg (αFRDg), FRDm2 (αFRDm2), or the three *T. brucei* FRD isoforms (αFRD). *E*, shows the analysis of the glycosomal localization of the FRD isoforms performed by *western blot* with the indicated immune sera on total trypanosome lysates (*left panel*) and purified glycosomes (*central panel*) of the parental and Δ*ppdk*/Δ*pepck*/^*RNAi*^GPDH.i cell lines. The glycosomal isocitrate dehydrogenase (IDHg) antibodies were used as a loading control. The *right panel* is a Coomassie staining of purified glycosomes, which highlights the absence of PPDK, PEPCK, GPDH, and FRDg in the glycosomes of the mutant cell line.

## Results

### Expression of a chimeric FRD isoform in the Δ*ppdk/*Δ*pepck/*^*RNAi*^GPDH mutant cell line

In order to study possible changes in gene expression of mutants missing key enzymes involved in the maintenance of the glycosomal redox and ATP/ADP balances, we have compared the total proteomes of the parental, Δ*ppdk* (24), Δ*pepck* (20), Δ*ppdk*/Δ*pepck* (18), and Δ*ppdk*/Δ*pepck*/^*RNAi*^GPDH.i (".i" stands for tetracycline-induced) cell lines, by label-free quantitative mass spectrometry. The effectiveness of this approach was confirmed by the 41.7–54.0-fold reduction observed for the PPDK and/or PEPCK peptide counts in the four mutant cell lines analyzed, compared with the parental cell line (see Fig. 2*A*, for the parental and Δ*ppdk*/Δ*pepck*/^*RNAi*^GPDH.i cell lines and the PXD020185 data set on the ProteomeXchange Consortium repository for the other cell lines). Similarly, the GPDH signal was strongly reduced (20.4-fold) in the Δ*ppdk*/Δ*pepck*/^*RNAi*^GPDH.i mutant. This analysis also showed that expressions of FRDg and FRDm2 were 6.9-fold decreased and tenfold increased, respectively, in the Δ*ppdk*/Δ*pepck*/^*RNAi*^GPDH.i cell line, while expression of FRDm1 was not affected (Fig. 2*A*). In contrast, expression of the three FRD isoforms remained unaffected in the three other mutant cell lines (PXD020185 data set on the ProteomeXchange Consortium). This FRD expression pattern was confirmed by western blotting using immune sera specific to FRDg (αFRDg) and FRDm2 (αFRDm2), in addition to the αFRD immune serum produced against the conserved FRDg central domain, which is 100% and 71% identical with FRDm2 and FRDm1, respectively (Fig. 2, *B* and *C*). The αFRD antibodies recognized two proteins in both the parental and Δ*ppdk*/Δ*pepck*/^*RNAi*^GPDH.i cell lines, including the ∼130 kDa FRDm1 isoform (Fig. 2, *D* and *E*). As previously reported, the second isoform expressed in the parental cell line (∼120 kDa) was recognized by the αFRDg, while no signal corresponding to FRDm2 was detected using αFRDm2 (24). In contrast, the ∼115 kDa protein expressed in the Δ*ppdk*/Δ*pepck*/^*RNAi*^GPDH.i cell line was recognized by αFRDm2, but not αFRDg. This suggests that the mutant cell line switched from FRDg to FRDm2 expression, although the apparent size of the detected FRDm2 isoform was higher than the theoretical one (∼115 *versus* 94.8 kDa). Coomassie staining, western blotting (Fig. 2*E*), and proteomic analyses (Fig. 2*A*) of purified glycosomal fractions confirmed the glycosomal localization of FRDg expressed in the parental cell line. In contrast, none of the FRD isoforms were detectable by western blot in the glycosomal fractions of the mutant cell line, which is consistent with the proteomic analyses.

**Figure 3:**
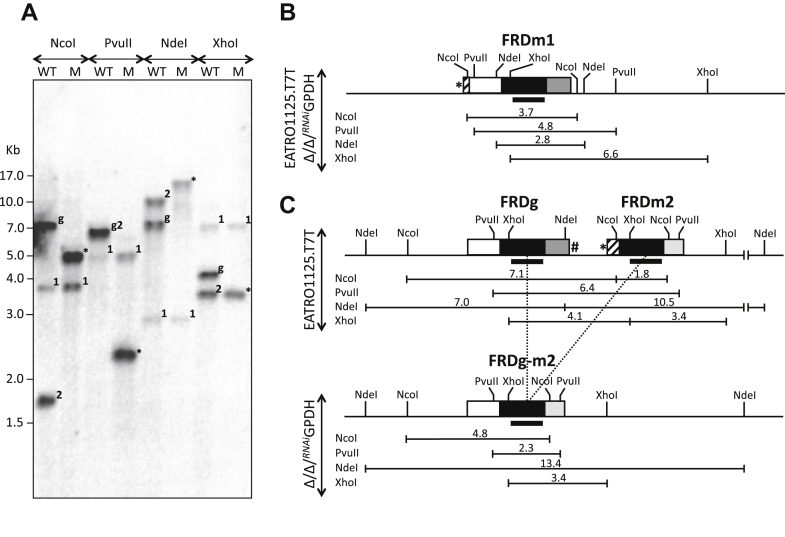
**Recombination inside the *FRDg*/*FRDm2* locus in the Δ*ppdk*/Δ*pepck*/**^***RNAi***^**GPDH cell line**. *A*, shows a Southern *blot* analysis of the parental (WT) and Δ*ppdk*/Δ*pepck*/^*RNAi*^GPDH mutant (M) genomic DNA after digestion with the NcoI, PvuII, NdeI, or XhoI restriction enzymes and probing with the *FRDg/FRDm2* central domain, homologous to the *FRDm1* gene (weak signals present in both cell lines). Abbreviations used to identify the labelled fragments: 1, FRDm1; 2, FRDm2; g, FRDg; ∗, FRDg-m2. The restriction maps presented in *panels* (*B*) (FRDm1) and the *upper part* of (*C*) (*FRDg/FRDm2* locus) are deduced from the genome sequence of the 927 strain available in TriTrypDB, while the lower part of (*C*) represents the FRDg/FRDm2 locus after deletion of the 4.1 kb fragment by homologous recombination (*dot lines*) in the Δ*ppdk*/Δ*pepck*/^*RNAi*^GPDH mutant cell line (Δ/Δ/^*RNAi*^GPDH). The size of the fragments is indicated in kb and the *black bars* represent the DNA fragment used to probe the *blot*. See Figure 2*B* legend for the color code of the genes.

To determine whether the mutually exclusive expression of FRDg and FRDm2 was related to genomic rearrangement inside the *FRDg*/*FRDm2* locus, a Southern blot analysis was conducted using as probe the conserved FRDg/FRDm2 central domain, which hybridizes with the *FRDm1* gene ("1" in Fig. 3*A*) but gives a much stronger signal for the *FRDg* and *FRDm2* genes ("g" and "2" in Fig. 3*A*). The restriction pattern obtained with the NcoI-, PvuII-, NdeI-, and XhoI-digested parental genomic DNA (Fig. 3*A*) was consistent with the restriction map of the FRDm1 (Fig. 3*B*) and FRDg/FRDm2 (Fig. 3*C*) loci deduced from the *T. brucei* TriTrypDB database (strain 927). Although the *FRDm1* locus was identical in the Δ*ppdk*/Δ*pepck*/^*RNAi*^GPDH genome, the pattern observed for the *FRDg*/*FRDm2* locus differed markedly (Fig. 3*A*). For instance, the 6.4 kb PvuII-fragment containing the two FRD genes in the parental genome was converted into a 2.3 kb PvuII-fragment in the mutant genome, suggesting that 4.1 kb had been deleted from the *FRDg/FRDm2* locus. Analysis of the three other restriction profiles led to the same conclusion. The size of the deleted DNA fragment (4.1 kb) was consistent with the size of the theoretical DNA fragment (4074 bp) resulting from homologous recombination between the central 1450 bp FRD domains, which are 100% identical in the *FRDg* and *FRDm2* genes.

In conclusion, these data showed that a recombination event occurred between the *FRDg* and *FRDm2* genes in the Δ*ppdk*/Δ*pepck*/^*RNAi*^GPDH mutant to generate a *FRDg-m2* chimeric gene coding for a FRD chimeric protein slightly smaller than FRDg (theoretical molecular weights: 120.6 *versus* 123.5 kDa, respectively). This DNA rearrangement event was present on both alleles of the locus, since the wild-type FRDg and FRDm2-amplified DNA fragments (Fig. 3*C*) and the endogenous FRDg protein (Fig. 2*D*) were not detectable in the mutant cell line.

### The homologous recombination event occurs in wild-type cells

**Figure 4:**
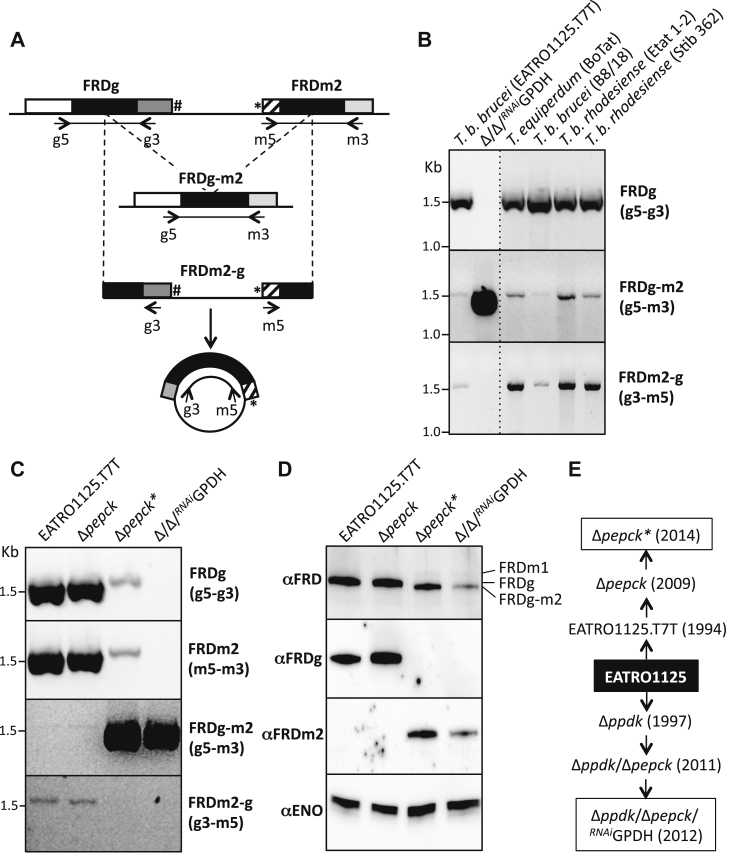
**PCR analysis of the recombination event in the *FRDg/FRDm2* locus**. The PCR strategy developed to detect a recombination event within the *FRDg/FRDm2* locus is described in (*A*). This schematic representation shows the DNA recombination event leading to the deletion of the FRDm2-g fragment, which can be circularized by ligation, as well as the position of the primers (*arrows*) designed to amplify the FRD domains of *FRDg* (g5-g3; 1583 bp), *FRDm2* (m5-m3; 1611 bp), and the chimeric *FRDg-m2* (g5-m3; 1577 bp), as well as the recircularized deleted FRDm2-g fragment (g3-m5; 1608 bp). See Figure 2*B* legend for the color code of the genes. *B*, shows a PCR analysis of the EATRO1125.T7T parental and Δ*ppdk*/Δ*pepck*/^*RNAi*^GPDH cell lines, as well as four additional African trypanosome strains, using 100 ng of genomic DNA (10 μg ml^−1^). In *panelsC* and *D*, a comparative analysis of the parental EATRO1125.T7T, Δ*pepck*∗, Δ*pepck*, and Δ*ppdk*/Δ*pepck*/^*RNAi*^GPDH cell lines is presented, using the PCR approach described in *panel A* (*C*) and the *western blot* analysis using antibodies specific for enolase (αENO), FRDg (αFRDg), FRDm2 (αFRDm2), or the three *T. brucei* FRD isoforms (αFRD) (*D*). The history of the Δ*ppdk*/Δ*pepck*/^*RNAi*^GPDH and Δ*pepck*∗ cell lines (*boxed*), which have selected the recombinant *FRDg*/*FRDm2* locus, is shown in (*E*). The Δ*pepck* cell line obtained in 2009 contains the parental *FRDg/FRDm2* locus, which became recombined later after long-term *in vitro* culture (Δ*pepck*∗ cell line, 2014).

To further study this DNA rearrangement event, we used PCR with primer pairs designed for amplification of the central FRD domain of the *FRDg* (g5 and g3 primers), *FRDm2* (m5 and m3 primers), and *FRDg-m2* (g5 and m3 primers) genes (see Fig. 4*A*). As expected, the FRDg- and FRDm2-specific DNA fragments were amplified from the parental EATRO1125.T7T cell line but not from the Δ*ppdk*/Δ*pepck*/^RNAi^GPDH genomic DNA (Fig. 4, *B* and *C*), confirming the loss of the wild-type *FRDg/FRDm2* locus in the mutant cell population. Also in agreement with the Southern blot data, the FRDg-m2-specific fragment was amplified from the mutant genomic DNA. Interestingly, however, the FRDg-m2-specific fragment was also very weakly PCR-amplified from the parental EATRO1125.T7T cell line, which suggests that the recombination event stochastically occurred in the wild-type cells (Fig. 4, *B* and *C*). Moreover, a 1.5 kb PCR product was obtained using parental DNA and the g3 and m5 primers; we suggest that the template was a circularized version of the deleted fragment (Fig. 4, *A*–*C*). No corresponding PCR product was detected in the mutant cell line, suggesting that the circularized deleted DNA fragment was not replicated and thus diluted during cell division to become undetectable. The same PCR analysis conducted on genomic DNA samples showed that the rearrangement event occurred in other strains of *T. brucei* (*Trypanosoma equiperdum*, *T. b. brucei*, and *T. b. rhodesiense*) (Fig. 4*B*).

**Figure 5:**
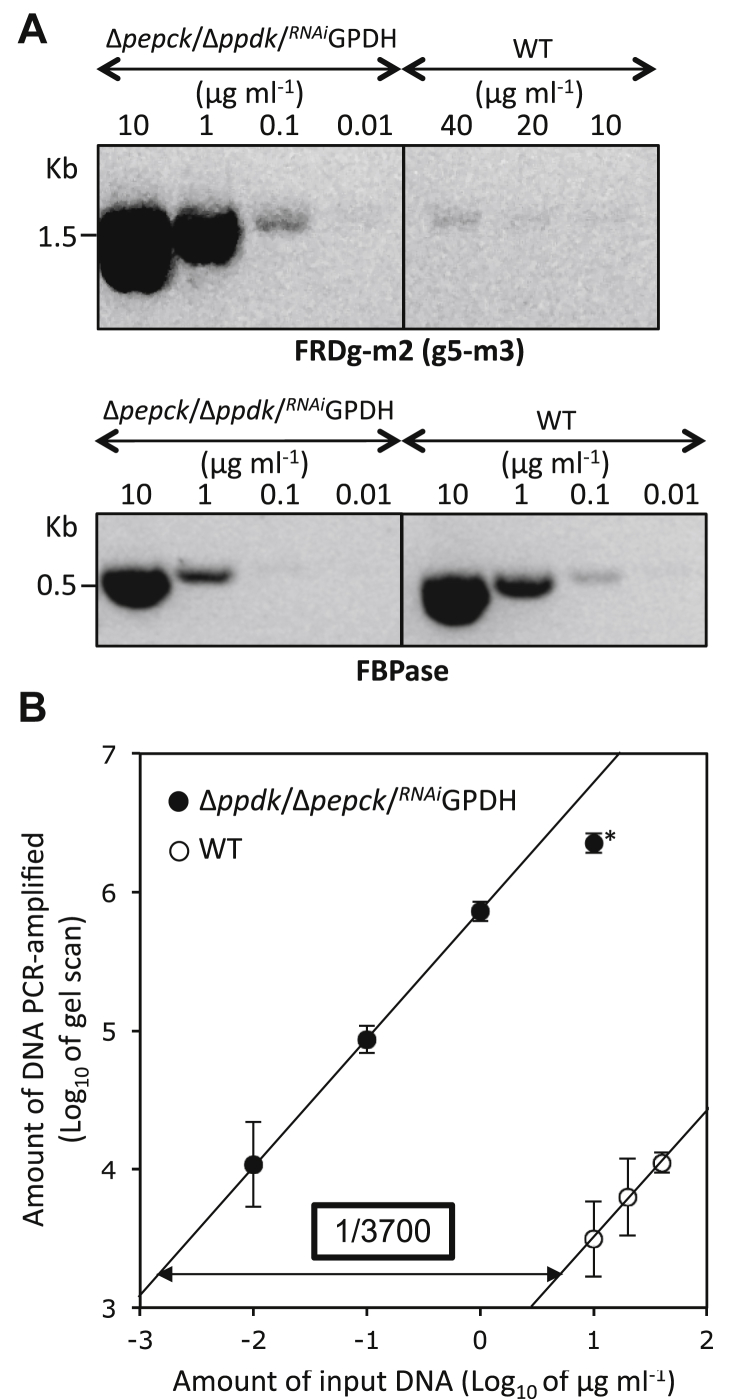
**Quantitation of recombined locus frequency**. *A*, shows PCR analyses of the parental (WT) and Δ*ppdk*/Δ*pepck*/^*RNAi*^GPDH cell lines using primers designed for DNA amplification of the chimeric *FRDg-m2* gene (*upper panel*) or the *FBPase* gene for normalisation (*lower panel*). The fraction of recombined loci was determined by calculating the relative difference between the two linear regressions of FRDg-m2 PCR signal (gel scan) as a function of the amount of input genomic DNA (*B*) (mean of three independent experiments). The value obtained for the FRDg-m2 PCR signal with 10 μg ml^−1^ of Δ*ppdk*/Δ*pepck*/^*RNAi*^GPDH genomic DNA, highlighted by an *asterisk*, was excluded from the trend *line*.

We took advantage of the Δ*ppdk*/Δ*pepck*/^*RNAi*^GPDH cell line being homozygous for the *FRDg-m2* recombinant locus to calculate the allele frequency of *FRDg-m2* in the EATRO1125.T7T parental cell line. We compared the *FRDg-m2* copy number in the two lines by semiquantitative PCR using different amounts of genomic DNA and the g5-m3 primer pair. Primers specific for a control gene (fructose-1,6-bisphosphatase, Tb927.9.8720) were used for normalization (Fig. 5*A*). The results showed that the *FRDg-m2* gene copy number is 3700-times higher in the Δ*ppdk*/Δ*pepck*/^*RNAi*^GPDH homogeneous cell line relative to the heterogeneous parental population (Fig. 5*B*), indicating that at the time of analysis one in 1850 cells in the parental population had a hemizygous recombined allele. This estimation is at best an upper limit since we cannot exclude PCR artifacts due to premature termination of the PCR product in the conserved region of one isoform followed by priming on the other isoform.

Altogether, this analysis suggested that the generation of the *FRDg-m2* chimeric gene occurs at low frequency by homologous recombination in the *T. brucei* genome, but was specifically selected in the Δ*ppdk*/Δ*pepck*/^*RNAi*^GPDH cell line.

### Selection of the *FRDg-m2* recombinant locus also occurred in the Δ*pepck* mutant cell line

Analysis of the Δ*pepck* mutant obtained and frozen in 2008 (20), then thawed in 2013, and maintained for weeks in culture (see Fig. 4*E*, here named Δ*pepck*∗), yielded more information about selection of the homologous recombination event in the *FRDg*/*FRDm2* locus. It is noteworthy that this Δ*ppdk*/Δ*pepck*/^*RNAi*^GPDH cell line was not derived from the Δ*pepck* cell line (see Fig. 4*E*). Indeed, the Δ*pepck* and the parental cells showed the same PCR profile, while the Δ*pepck∗* cells maintained for a long term in *in vitro* culture showed a pattern similar to the Δ*ppdk*/Δ*pepck*/^*RNAi*^GPDH cell line. Therefore, selection of the recombinant allele occurred independently in a second mutant cell line (Fig. 4*C*). However, the selection process was probably less stringent or more recent in the *PEPCK* null background compared with the Δ*ppdk*/Δ*pepck*/^*RNAi*^GPDH background, as illustrated by the presence of the wild-type locus in the Δ*pepck*∗ population (g5-g3 and m5-m3 primer pairs in Fig. 4*C*), even after months of growth. As expected from the PCR analysis, the FRDg-m2 chimeric isoform was expressed in the Δ*pepck*∗ cell line, but not detectable by western blotting in the Δ*pepck* cells (Fig. 4*D*). It is noteworthy that proteomics analysis performed on the Δ*pepck* cell line before long-term cultivation showed an intermediate profile of FRD isoform expression between the parental and the Δ*ppdk*/Δ*pepck*/^*RNAi*^GPDH cell lines (PXD020185 data set on the ProteomeXchange Consortium). These data strongly suggest selection for the *FRDg-m2* recombinant locus when *PEPCK* is missing (Fig. 4*E*).

### Cytosolic localization of the inactive chimeric FRDg-m2 isoform

**Figure 6:**
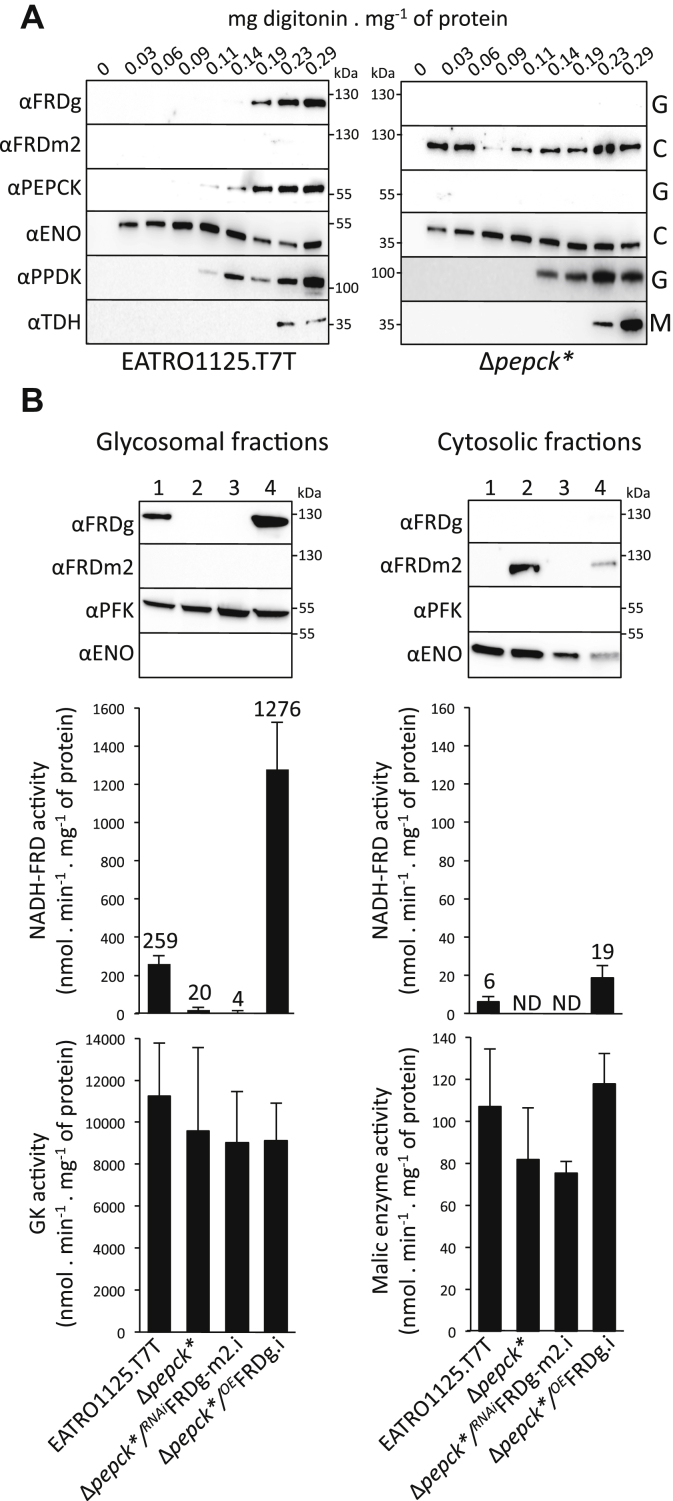
**The cytosolic FRDg-m2 chimeric isoform is not enzymatically active**. *A*, shows the glycosomal and cytosolic localization of FRDg and the chimeric FRDg-m2 isoforms, respectively, by digitonin titration. The supernatant collected from the EATRO1125.T7T and Δ*pepck*∗ cells incubated with 0–0.29 mg of digitonin per mg of protein was analyzed by *western blot* using the anti-FRDg, anti-FRDm2 as well as immune sera against cytosolic (enolase, αENO), glycosomal (αPPDK), and mitochondrial (threonine dehydrogenase, αTDH) markers. *B*, shows the *western blotting* and enzymatic activities determined in the glycosomal and cytosolic fractions of EATRO1125.T7T (1), Δ*pepck*∗ (2), Δ*pepck*∗/^*RNAi*^FRDg-m2.i (3), and Δ*pepck*∗/^*OE*^FRDg.i (4) cells lines. Expression of FRDg and the chimeric FRDg-m2 isoforms was determined by *western blotting* using the anti-FRDg and anti-FRDm2 immune sera (*top panel*). Immune sera against the glycosomal phosphofructokinase (αPFK) and the cytosol enolase (αENO) were used as loading controls. NADH-FRD activity was determined on the same fractions used for *western blot* analyses (mean of three independent experiments). For normalization, the glycerol kinase (GK) and malic enzyme activities were also determined in the glycosomal and cytosolic fractions (*lower panel*).

The glycosomal localization of FRDg was confirmed by a digitonin cell fractionation experiment. Western blot analysis of the supernatant fractions confirmed that as expected, the FRDg isoform was released together with the PPDK and PEPCK glycosomal markers (Fig. 6*A*). In contrast, the FRDg-m2 chimeric isoform expressed in the Δ*pepck*∗ cell line was released at lower digitonin concentrations (0.03 mg *versus* 0.14 mg of digitonin per mg of protein) together with the enolase cytosolic marker (Fig. 6*A*). The cytosolic location of the FRDg-m2 chimeric isoform expressed in the Δ*pepck*∗ cell line was confirmed by a western blot analysis of glycosomal and cytosolic fractions prepared by differential centrifugation after silicon carbide cell homogenization (Fig. 6*B*).

The NADH-dependent FRD (NADH-FRD) activity was determined in the glycosomal and cytosolic fractions of the original EATRO1125.T7T and the Δ*pepck*∗ cell lines. As expected, NADH-FRD activity was detected in the glycosomal fraction of cells expressing FRDg (EATRO1125.T7T), but not in the glycosomes of the Δ*pepck∗* cell line (Fig. 6*B*). The low level of NADH-FRD activity detected in the cytosolic fraction of the EATRO1125.T7T cell line, compared with the glycosomal fraction (2.3%), was presumably due to the lysis of a few glycosomes during the grinding step. The absence of NADH-FRD activity in the cytosolic fraction of the Δ*pepck*∗ cell line demonstrates that the chimeric FRDg-m2 isoform is inactive (Fig. 6*B*). These data highlight the role of the Cytb5R domain in the NADH-FRD activity, since only this domain differs between the active FRDg and inactive FRDg-m2 isoforms.

### Expression of FRDg affects the Δ*pepck*∗ growth rate

**Figure 7:**
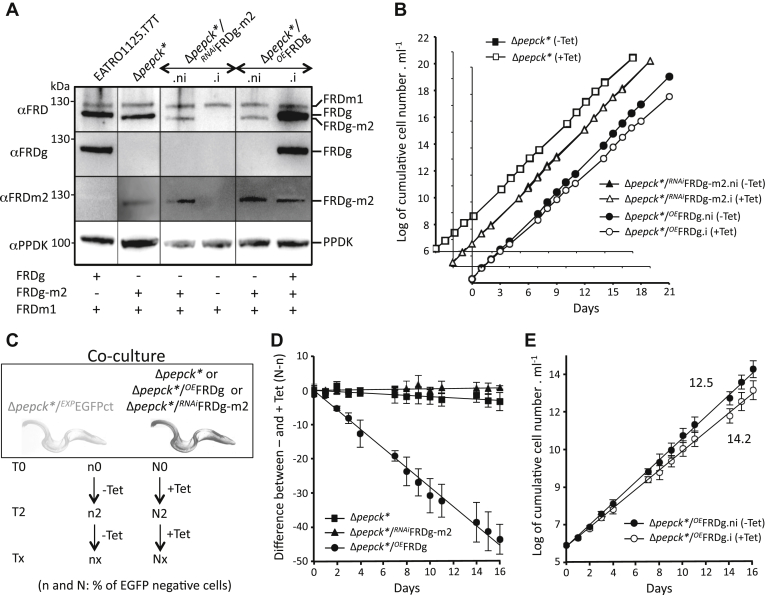
**Expression of FRDg is responsible for reduced growth of the Δ*pepck*∗ cell line.***A*, expression of the FRD isoforms in the EATRO1125.T7T and Δ*pepck*∗ parental cell lines, as well as the tetracycline-induced (.i) or noninduced (.ni) Δ*pepck*∗/^*RNAi*^FRDg-m2 and Δ*pepck*∗/^*OE*^FRDg was monitored by *western blot* analysis using immune sera indicated on the *left margin*. Expression of the FRD isoform(s) is indicated under the *blot*. The growth curves of these tetracycline-induced (.i or +Tet) or noninduced (.ni or -Tet) cell lines are shown in (*B*). To confirm the moderate growth defect observed for the Δ*pepck*∗ mutant expressing the recombinant FRDg (Δ*pepck*∗/^*OE*^FRDg.i), the Δ*pepck*∗, Δ*pepck*∗/^*RNAi*^FRDg-m2, and Δ*pepck*∗/^*OE*^FRDg cell lines were co-cultured with the Δ*pepck*∗ cell line constitutively expressing EGFP (Δ*pepck*∗/^*OE*^EGFPct), in the presence or the absence of *tetracycline*. Flow cytometry analyses were conducted to determine EGFP positive cells (Δ*pepck*∗/^*OE*^EGFPct) and EGFP negative cells (Δ*pepck*∗ or double mutant cell lines) all along the growth curve, as illustrated in (*C*). *D*, shows the difference of the percentage of EGFP negative cells between noninduced and induced conditions, all along the 16-day co-culture (mean of three independent experiments). The growth curve of induced and noninduced Δ*pepck*∗/^*OE*^FRDg co-cultured with the EGFP-tagged parental cell line (Δ*pepck*∗/^*OE*^EGFPct) was deduced from the same data sets and plotted in panel E. The numbers indicate the population doubling time in both growth conditions.

Selection of the *FRDg-m2* recombinant locus in the Δ*pepck∗* and Δ*ppdk*/Δ*pepck*/^*RNAi*^GPDH cell lines implied that either expression of the FRDg-m2 isoform in the cytosol or abolition of FRDg expression in the glycosomes provided a selective advantage to both mutant cell lines. To determine which of these two hypotheses is correct, tetracycline-inducible ectopic expression of FRDg and RNAi-mediated downregulation of FRDg-m2 were performed in the Δ*pepck∗* cell line (Δ*pepck∗*/^*OE*^FRDg and Δ*pepck∗*/^*RNAi*^FRDg-m2, respectively) (Fig. 7*A*). These experiments could not be conducted with the Δ*ppdk*/Δ*pepck*/^*RNAi*^GPDH cell line, because all five available selectable markers had already been used. The glycosomal localization of the recombinant FRDg in the Δ*pepck∗*/^*OE*^FRDg.i line was confirmed by western blotting and enzymatic activity assay of glycosomal fractions (Fig. 6*B*). The doubling time of the Δ*pepck*∗/^*RNAi*^FRDg-m2 cell population was identical in the absence (.ni) or the presence (.i) of tetracycline, indicating that expression of the FRDg-m2 chimera was well tolerated by the Δ*pepck*∗ mutant. In contrast, induction of FRDg expression slightly reduced the growth rate of the Δ*pepck*∗/^*OE*^FRDg.i cell line (Fig. 7*B*).

Selection against FRDg expression was confirmed in a co-culture experiment. EGFP-tagged Δ*pepck*∗ cell line (Δ*pepck*∗/^*OE*^EGFPct, constitutively expressing EGFP) was co-cultured with the Δ*pepck*∗ (control), Δ*pepck*∗/^*RNAi*^FRDg-m2 or Δ*pepck*∗/^*OE*^FRDg cell lines in the presence or the absence of tetracycline, and the proportion of EGFP-positive cells was determined over time by flow cytometry (Fig. 7*C*). The percentage of EGFP negative cells between induced and noninduced conditions gradually decreased when FRDg was expressed in the Δ*pepck*∗ cell line, while there was no selection against FRDg-m2 expression (Fig. 7*D*). Calculations revealed that expression of FRDg increased the doubling time of the Δ*pepck*∗ cells from 12.5 h to 14.2 h (Fig. 7*E*).

**Figure 8:**
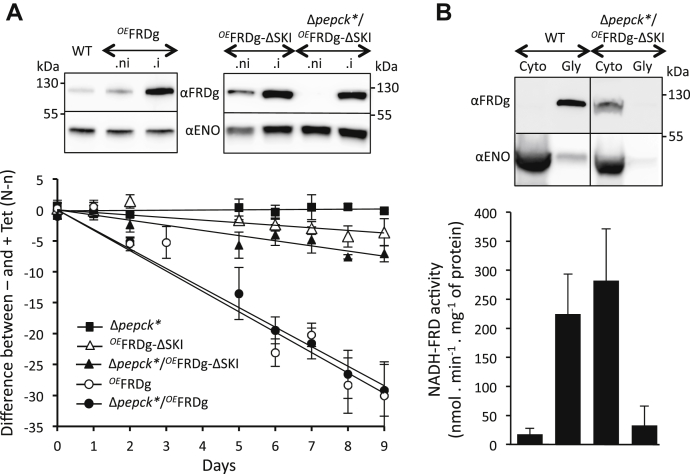
**The cytosolic expression of FRDg does not affect growth of the Δ*pepck*∗ cell line.***A*, shows the effect of the expression of full length FRDg and FRDg-ΔSKI in the WT (^*OE*^FRDg and ^*OE*^FRDg-ΔSKI) or the Δ*pepck*∗ background (Δ*pepck*∗/^*OE*^FRDg and Δ*pepck*∗/^*OE*^FRDg-ΔSKI) using as control the Δ*pepck*∗ cell line. For this experiment, the mutant cell lines were co-cultured with the Δ*pepck*∗ cell line constitutively expressing EGFP (Δ*pepck*∗/^*OE*^EGFPct), in the presence or the absence of tetracycline, as described in Figure 7*C*. The difference of the percentage of EGFP negative cells between noninduced and induced conditions is plotted as a function of time of growth (mean of three independent experiments). The *top panel* shows a *western blot* analysis of these tetracycline-induced (.i) or noninduced (.ni) cell lines. *B*, expression of the endogenous FRDg isoform in the parental EATRO1125.T7T cell line (WT) or the recombinant FRDg-ΔSKI in the tetracycline-induced Δ*pepck*∗/^*OE*^FRDg-ΔSKI mutant was monitored in the glycosomal (Gly) and cytosolic (Cyto) fractions by *western blot* analysis using the immune sera indicated on the *left margin*. The *lower panel* shows the glycosomal and cytosolic NADH-FRD activities normalized with the GK and malic enzyme activities, respectively (mean of five independent experiments).

Recombinant FRDg was ∼4.5-times more expressed in the Δ*pepck*∗/^*OE*^FRDg.i cell line than the endogenous FRDg in the parental WT cells (Fig. 6*B*). Interestingly, overexpression of FRDg in the WT background (^*OE*^FRDg.i) also induced a significant growth defect (Fig. 8*A*). FRDg overexpression is therefore detrimental in the absence as well as in the presence of PEPCK. This does not, however, affect our overall conclusions: the native levels of FRDg expression must affect the Δ*pepck* and Δ*ppdk*/Δ*pepck*/^*RNAi*^GPDH cell lines more than the EATRO1125.T7T parental cell line, since the recombined *FRDg*/*FRDm2* locus has been positively selected in the mutant cell lines.

### The glycosomal localization of FRDg is required for the growth retardation

We next tested whether the negative effect of FRDg expression in the Δ*pepck∗* background was dependent on the glycosomal localization of the protein. To address this question, the C-terminal peroxisomal targeting signal (PTS1) composed of the last three amino acids, the SKI tripeptide in FRDg, was removed from the recombinant protein to express a functional FRD in the cytosol of the Δ*pepck*∗ cell line (Δ*pepck*∗/^*OE*^FRDg-ΔSKI). The cytosolic localization of the FRDg-ΔSKI was confirmed by western blotting of the glycosomal and cytosolic fractions and NADH-FRD activity was detected in the cytosolic fraction of the Δ*pepck*∗/^*OE*^FRDg-ΔSKI.i cell line (Fig. 8*B*). Co-culture of the Δ*pepck*∗/^*OE*^EGFPct cell line with the Δ*pepck*∗/^*OE*^FRDg-ΔSKI or ^*OE*^FRDg-ΔSKI mutants in the presence or the absence of tetracycline, performed as above, revealed only minimal growth retardation upon FRDg-ΔSKI expression in the Δ*pepck*∗ or WT backgrounds (Fig. 8*A*). Thus the glycosomal expression of FRDg was required to affect growth of the Δ*pepck*∗ cell line.

### The glycosomal NADH-FRD activity is not responsible for the growth retardation

**Figure 9:**
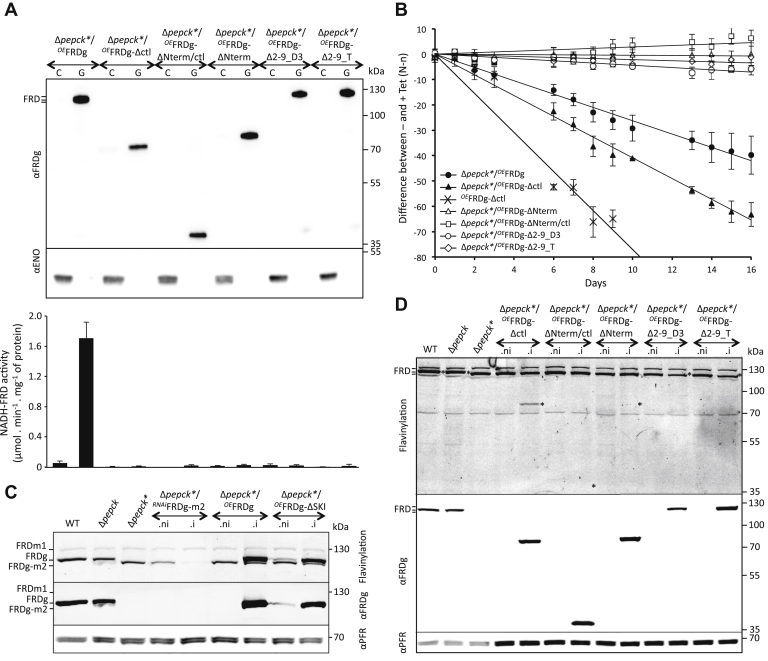
**The flavinylation motif of the FRDg N-terminal domain is required for growth retardation of the Δ*pepck*∗ cell line.***A*, shows *western blot* analyses of cytosol-enriched (C) and glycosome-enriched (G) fractions from Δ*pepck∗* cell lines expressing truncated or mutated recombinant FRDg. See Figure 6 for the immune sera used. The *lower panel* shows the glycosomal and cytosolic NADH-FRD activities normalized with the GK and malic enzyme activities, respectively (mean of three independent experiments). *B*, shows the effect of the expression of FRDg, FRDg-Δctl, FRDg-ΔNterm, FRDg-ΔNterm/ctl, and FRDg-Δ2-9 (clones D3 and T) in the Δ*pepck*∗ background and expression of FRDg-Δctl in the parental EATRO1125.T7T background (*^OE^*FRDg-Δctl), as described in Figures 7 and 8. The *western blot* control of the ^*OE*^FRDg-Δctl cell line is shown in Figure S1. The difference of the percentage of EGFP negative cells between noninduced and induced conditions is plotted as a function of time of growth (mean of three independent experiments). *C* and *D*, show the presence of covalent flavinylation of the FRD isoforms and mutants in the cell lines analyzed (.i, tetracycline-induced; .ni, noninduced). The *top panels* show directly detected fluorescence of covalently bound flavin on a denaturing gel, while the *lower panels* show *western blot* analyses with the anti-FRDg and anti-PFR (internal loading reference) immune sera. In *D*, the locations of the endogenous and recombinant FRDg protein bands are indicated by an *asterisk* (∗) on the direct fluorescence gel image; the positions of FRD isoform bands are indicated at the left gel margin, as detailed in (*C*).

FRDg is composed of an N-terminal ApbE-like domain, a central fumarate reductase domain (FRD), and a C-terminal cytochrome b5 reductase domain (Cytb5R) (Fig. 2*B*). To determine the FRDg domains responsible for the negative growth effect of the FRDg in the Δ*pepck* background, truncated recombinant FRDg proteins missing the FRD (Δctl), ApbE-like (ΔNterm) or ApbE-like/FRD (ΔNterm/ctl) domains were expressed in the Δ*pepck∗* cell line (Δ*pepck∗*/^*OE*^FRDg-Δctl, Δ*pepck∗*/^*OE*^FRDg-ΔNterm, and Δ*pepck∗*/^*OE*^FRDg-ΔNterm/ctl, respectively). All three recombinant FRDg proteins were successfully expressed in the glycosomes, and as expected none of them showed NADH-FRD activity (Fig. 9*A*). The Cytb5R domain alone (FRDg-ΔNterm/ctl) or in combination with the FRD domain (FRDg-ΔNterm) did not affect growth, but surprisingly, expression of FRDg-Δctl was even more deleterious than full-length FRDg (Fig. 9*B*). Clearly, specific NADH-FRD enzymatic activity was not responsible for growth retardation (Fig. 9*B*). Overexpression of recombinant ^*OE*^FRDg-Δctl also inhibited growth of the EATRO1125.T7T parental cell line.

### Growth retardation depends on flavinylation

The absence of an altered growth phenotype upon expression of FRDg-ΔNterm suggested a possible role of the ApbE-like domain. Recently, Serebryakova *et al.* (23) showed that the orthologous FRDg of *L. pyrrhocoris*, a trypanosomatid related to trypanosomes, contains a covalently attached flavin at serine 9 of the N-terminal flavinylation motif [D_3_(g/s)x(s/t)(s/g)AS_9_]. They suggested that the ApbE-like domain may catalyze the transfer of FMN from FAD to serine 9 of FRDg. Replacement of S9 by an asparagine residue abolished both flavinylation and NADH-fumarate reductase activity of *Leptomonas* FRDg. We therefore addressed the role of flavinylation for FRD activity in trypanosomes and for the growth phenotype caused by glycosomal expression of FRDg in the Δ*pepck∗* background. The FRDg-Δ2-9 mutant protein missing the first nine N-terminal residues (the suggested flavinylation motif), expressed in the Δ*pepck∗* background (Δ*pepck∗*/^*OE*^FRDg-Δ2-9_D3 and Δ*pepck∗*/^*OE*^FRDg-Δ2-9_T cell lines) did not confer glycosomal FRD activity (Fig. 9*A*) upon tetracycline-induced expression and caused no growth retardation (Fig. 9*B*).

**Figure 10:**
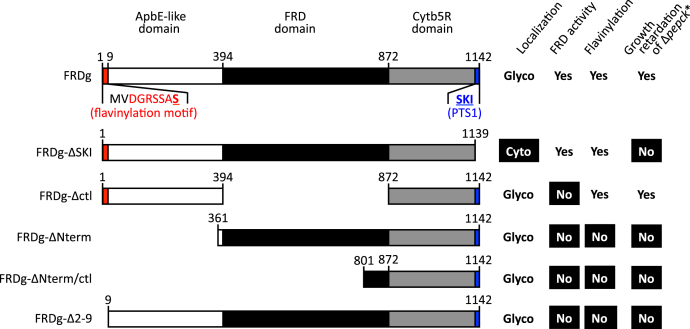
**Correlations between FRD domains, FRD flavinylation, glycosomal localization, and effect on growth of the Δ*pepck*∗ cell line.** This figure summarizes expression of the endogenous and mutated FRDg in the Δ*pepck*∗ background, their subcellular localization, NADH-dependent FRD activity, covalent flavinylation, and the effect on growth of the Δ*pepck*∗ cell line. The *white, black*, and *gray boxes* represent the ApbE-like, fumarate reductase, and cytochrome *b*_5_ reductase domains, respectively, and the consensus flavinylation motif is indicated in red (the serine residue that is most likely the covalent attachment site of the flavin moiety is *bold* and *underlined*). Difference of phenotype compared with the Δ*pepck∗*/^*OE*^FRDg (FRDg) cell line expressing endogenous FRDg is indicated in *white* on a *black background.* Glyco, glycosome; Cyto, cytosol.

The flavinylation of all endogenous and expressed FRD isoforms and mutants was directly assessed by in-gel detection of flavin fluorescence at 526 nm. Using denaturing gels and boiling of protein samples, only covalently linked flavin was detected (25) (Fig. 9, *C* and *D*). For the glycosomally expressed FRD mutant proteins, flavinylation correlates with growth phenotype (Fig. 10). This confirmed that covalent flavinylation is required for FRD activity *in vivo* in trypanosomes (Fig. 9*A*). The essential role of FRD flavinylation for the altered growth phenotype implicates electron transfer, albeit not to fumarate, in the deleterious effects.

### Absence of significant FRDg-catalyzed flux in the Δ*pepck* mutant

Why does FRDg impair growth of the Δ*pepck* cell line? FAD-containing enzymes, including FRD, are known to transfer electrons very efficiently to oxygen to generate toxic ROS. We therefore hypothesized that in the absence of metabolic flux through the glycosomal succinate branch, the decrease of fumarate allows oxygen to become the main FRDg substrate. To determine the contribution of FRDg to the production of succinate, we analyzed excreted end products (exometabolome) from the metabolism of the two main carbon sources used by the parental and mutant cell lines (glucose and proline) using the ^1^H-NMR profiling approach. The advantage of ^1^H-NMR spectrometry is the possibility to distinguish protons bound to ^12^C and ^13^C carbons, so that end products excreted from two different carbon sources can be distinguished, provided that one is uniformly ^13^C-enriched. To achieve this, we used [U-^13^C]-glucose and nonenriched proline (26). The parental trypanosomes incubated in the presence of glucose and proline produced 71% acetate, 18% succinate, and 11% alanine as excreted end products (Table 1). As expected, succinate was no longer produced from glucose in the Δ*pepck* cell line (20), while the amounts of proline-derived succinate were not affected (see Fig. 1). The metabolic patterns of the Δ*pepck* and Δ*pepck∗* cell lines were similar, indicating that succinate excreted from proline in the PEPCK null background was produced not in the glycosomes, but in the mitochondrion by succinyl-CoA synthetase as previously proposed (26). Our interpretation of these data is consistent with the maintenance of the level of succinate production from proline in the Δ*pepck∗*/^*OE*^FRDg.i mutant, in which expression of FRDg is 4.5 times higher than in the EATRO1125.T7T cell line (WT) (Table 1). As expected, no difference was also observed in the Δ*pepck∗*/^*RNAi*^FRDg-m2.i (Table 1). These data suggest that the observed growth phenotype is probably the consequence of the absence of succinate production within the glycosomes, which may lead to an increased production of ROS by FRDg. An equivalent NMR experiment was conducted on the Δ*pepck∗*/^*OE*^FRDg-ΔSKI cell line in the presence of 4 mM nonenriched glucose and [U-^13^C]-proline. As expected, the amounts of ^13^C-enriched-succinate excreted from [U-^13^C]-proline are unchanged before and after induction of FRDg-ΔSKI expression (Table S1).

**Table 1:** Excreted end products from metabolism of [U-^13^C]-glucose and nonenriched proline by BSF parental and mutant cell lines

Excreted end products (carbon source)	WT	Δ*pepck*	Δ*pepck∗*	Δ*pepck∗*/^*OE*^FRDg.ni^a^	Δ*pepck∗*/^*OE*^FRDg.i^a^	Δ*pepck∗*/^*RNAi*^FRDg-m2.ni	Δ*pepck∗*/^*RNAi*^FRDg-m2.i
	nmol·h^−1^·10^8^ cells^−1^^b^						
n^c^	3	3	3	3	3	3	3
Succinate (glucose^d^)	277 ± 15	ND	ND	ND	ND	ND	ND
Succinate (proline^d^)	132 ± 19	147 ± 21	138 ± 14	94 ± 7	78 ± 6	78 ± 4	72 ± 4
Acetate (glucose)	1431 ± 121	385 ± 25	400 ± 39	414 ± 32	409 ± 10	402 ± 20	394 ± 11
Acetate (proline)	238 ± 12	366 ± 26	395 ± 39	308 ± 16	330 ± 18	345 ± 19	347 ± 15
Alanine (glucose)	229 ± 25	374 ± 29	405 ± 41	367 ± 23	391 ± 13	409 ± 14	400 ± 7.
Alanine (proline)	31 ± 4	142 ± 11	156 ± 20	163 ± 8	182 ± 15	140 ± 15	141 ± 8.
Total (glucose)	1950 ± 120	759 ± 54	806 ± 78	781 ± 54	800 ± 12	811 ± 34	794 ± 16
Total (proline)	401 ± 15	655 ± 56	689 ± 71	565 ± 29	590 ± 15	563 ± 28	561 ± 20

ND, not detectable.

a i: RNAi cell line induced during 5 days by addition of tetracycline;.ni: noninduced RNAi cell line.

b The amounts of end products excreted from glucose and proline metabolism are expressed as nmoles excreted per hour and per 10^8^ cells.

c Number of biological replicates.

d Carbon source metabolized into succinate.

### Growth retardation is correlated with increased ROS production

**Figure 11:**
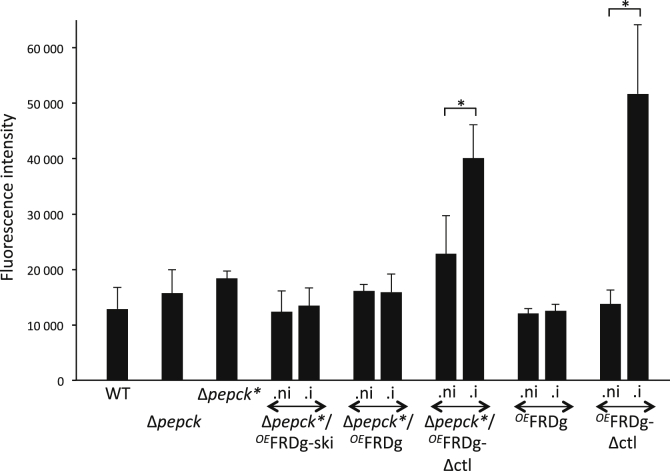
**FRDg-Δclt-mediated increase of ROS production.** The cellular ROS detection reagent H_2_DCFDA was quantified by fluorimetry (means ± SD, n = 3 independent experiments, ∗*p* value < 0.05) for the parental and mutant cell lines grown in glucose-rich conditions.

To test our hypothesis that production of ROS by FRDg is responsible for the observed growth retardation, ROS production was measured in the respective cell lines. To do so, we used the H_2_DCFHDA probe, which enters all subcellular compartments before being oxidized to a green fluorescent compound by hydrogen peroxide. The two cell lines showing the largest growth reduction, *i.e*., Δ*pepck∗*/^*OE*^FRDg-Δctl.i and ^*OE*^FRDg-Δctl.i, produce 1.8 and 3.7 times more ROS, respectively, than the uninduced cell lines, which is consistent with our hypothesis (Fig. 11). The observation that ROS production is not increased in the Δ*pepck∗*/^*OE*^FRDg.i and ^*OE*^FRDg.i cell lines is interpreted as a limitation of the whole-cell ROS quantification method. These lines with only moderate growth retardation are expected to have low ROS production that remains below the assay detection limit. As expected, the amounts of ROS were also unchanged upon induction of FRDg-ΔSKI expression (Fig. 11).

## Discussion

### Stochastic recombination in *T. brucei*

Stochastic recombination leading to amplification of chromosomal regions located between homologous direct or inverted repeated sequences has been observed in *Leishmania major* (2). This genome-wide phenomenon leads to extrachromosomal circular or linear amplified DNAs, as well as deletion of DNA fragments. DNA amplification through stochastic recombination has a direct impact on gene dosage and fosters the selection of adaptive traits in response to environmental pressure, such as drug exposure, as previously reported on many occasions (27). However, the benefit provided by deletion of DNA fragments is much less obvious. In contrast to *Leishmania* spp., the involvement of DNA amplification by stochastic recombination in adaptation to environmental pressure has not been reported so far for *T. brucei*, presumably due to a stricter replication system where episomal vector maintenance is the exception (28). This implies that gene deletions and generation of mosaic genes are the observable effects of stochastic recombination in *T. brucei* (11, 29, 30). Δ*pepck* cell lines expressing the chimeric FRDg-m2 instead of FRDg constitute one of the two examples of a stochastic recombination event providing a selective advantage to *T. brucei* reported to date. The other example has been described in the tandemly arranged genes encoding aquaglyceroporins (AQP2 and AQP3), which facilitate the transmembrane transport of water and small nonionic solutes. In *T. brucei*, AQP3 plays a role in the osmoregulation and transport of glycerol, while AQP2 is also a carrier involved in importing the trypanocidal melarsoprol and pentamidine drugs by endocytosis (11, 31, 32). In some melarsoprol/pentamidine-resistant cell lines, the *AQP2* and *AQP3* genes are replaced by a chimeric *AQP2/3* gene, which lost the capacity to interact with the drugs (11, 30). Since the *AQP2* (Tb927.10.14170) and *AQP3* (Tb927.10.14180) genes are 80% identical, with three ∼80 bp direct repeats, and are only separated by 857 bp of noncoding sequence, formation of the *AQP2/3* chimeric gene most probably results from drug selection of stochastic homologous recombination within the *AQP2*/*AQP3* locus, as described here for the *FRDg*/*FRDm2* locus.

### The C-terminal Cytb5R domain is involved in FRD activity

The gene encoding FRDg is composed of three domains, the central FRD domain (433 aa) flanked by the C-terminal Cytb5R domain (222 aa) and the N-terminal ApbE-like flavin transferase domain (287 aa) (21, 22, 23). FRDg is responsible for the glycosomal NADH-dependent FRD activity; however, the role of the N- and C-terminal domains is currently unknown. Here, we provide evidence that the Cytb5R domain is required for the FRD activity, since the FRDg-m2 chimera expressed in the cytosol of the Δ*pepck* cell line is not active. Indeed, the only difference between the endogenous FRDg and chimeric FRDg-m2 isoforms is the C-terminal domain showing 34% (e-value: 9 × 10^−32^) and 27% (evalue: 3 × 10^−8^) identity with the *Cryptococcus neoformans* Cytb5R, respectively. Since, the NADH-cytochrome b5 reductases are known to transfer electrons from NADH to cytochrome b5 (33), one may consider that the Cytb5R domain is part of the electron transfer channel required for the FRD activity. Elucidating the 3D structure of FRDg is required to define the mechanism of FRDg activity and characterize this electron transfer channel.

### Role of FRDm2

Our data suggest that the FRDm2 isoform has lost FRD activity, since it shares the C-terminal domain of the inactive FRDg-m2 chimera and more importantly it lacks the N-terminal ApbE-like domain including the flavinylation motif (see Fig. 2*C*, (22)). The *FRDg*/*FRDm2* locus is conserved across the trypanosomatid lineage, with the FRD/Cytb5R composite structure of the FRDm2 isoforms conserved within the *Trypanosoma* species, while *Crithidia fasciculata* and the *Leishmania* spp. have lost the C-terminal Cytb5R domain. The conservation of FRDm2 in the *Trypanosoma* and *Leishmania* branches, which separated 400–600 millions years ago (34), is surprising as expression of the FRDm2 isoform was neither detectable in the PCF nor in bloodstream forms of *T. brucei* (22) and absence of FRD-specific activity, leaving a biological function enigmatic. Perhaps FRDm2 has a role in stages that have yet to be examined in detail, such as the epimastigotes.

### Overexpression of FRDg also affects growth of the parental cell line

Selection of the FRDg-m2 chimera in the Δ*pepck* and Δ*ppdk*/Δ*pepck*/^*RNAi*^GPDH cell lines implies that this stochastic recombination event was beneficial for the PCF trypanosomes in the context of the PEPCK null background. We demonstrated that this selection is driven by the deleterious effect of FRDg overexpression in the PEPCK null background, which provided a rational explanation for the selection of the recombined Δ*pepck∗* cells. It is noteworthy that a ∼5-fold overexpression of FRDg also slightly affected growth of the parental EATRO1125.T7T cells, suggesting that the normal FRDg level is compatible with—and perhaps supports—optimal growth of the wild-type parasite, whereas it impairs growth of the Δ*pepck* mutant. This hypothesis may explain why the recombination event has been selected in the PEPCK null background but not in the wild-type background.

### How to explain the growth phenotype due to overexpression of FRDg?

Several observations support the view that FRDg, as already observed for FRD from other organisms, can produce ROS, known to be toxic at high concentrations. For instance, FRD is a major contributor to ROS formation in *Bacteroides fragilis* exposed to oxygen (35). ROS are formed by autoxidation when redox enzymes accidentally transfer electrons to oxygen rather than to their physiological substrates. This enzymatic promiscuity is well illustrated by the *Escherichia coli* aspartate:fumarate oxidoreductase, which was conventionally named aspartate oxidase since oxygen is used as electron acceptor in aerobic conditions (36). However, this enzyme can also transfer electrons to fumarate, which is certainly its natural substrate in the anaerobic conditions encountered in the intestine by *E. coli* (37). This autoxidation activity of FAD-dependent redox enzymes is due to the solvent accessibility of the flavin moiety, which is situated at the protein surface in order to interact with soluble substrates, as described for the *E. coli* FRD in the absence of its natural substrate, *i.e.* fumarate (38). The notion that oxygen and fumarate compete for electrons provided by FAD was also reported for the *T. brucei* FRD since fumarate inhibited hydrogen peroxide formation with the same affinity as it stimulated NADH-dependent FRD activity (K_*i*_ = 16 *versus* 20 μM) (39). Thus, abolition of glycosomal succinate production in the Δ*pepck* background, which is probably due to a significant reduction of the glycosomal amounts of fumarate (see Fig. 1), might stimulate autoxidation activity of FRDg. This hypothesis is consistent with the absence of growth phenotype for the Δ*pepck∗*/^*OE*^FRDg-Δ2-9 cell lines, which lost the covalently bound flavin required to transfer electron to the acceptor (Fig. 9*B*). More importantly, overexpression of the recombinant FRDg-Δctl in Δ*pepck∗* background, as well as in the wild-type cells, induces a significant increase of ROS production, which is most probably responsible for the associate growth retardation. These data are consistent with the proposed competition between fumarate and oxygen for electrons provided by the covalently bound flavin in the ApbE domain. Indeed, in the absence of the central FRD domain, oxygen would become the main electron acceptor, regardless of the amounts of fumarate inside the glycosomes. It is noteworthy that overexpression of a cytosolic version of FRDg does not affect growth of the Δ*pepck∗* and parental cell lines, unlike glycosomal FRDg, which is probably due to the higher oxidative stress defense capacity of the cytosol *versus* the glycosomes.

### What would be the possible role of ROS production by the glycosomal fumarate reductase?

ROS have historically been viewed as toxic metabolic by-products and causal agents of many pathologies. This notion is indeed supported by irreversible damages to cellular components caused by high levels of cellular ROS (40). However, this line of thinking has gradually shifted toward a more positive view with the growing body of evidence showing that lower levels of ROS are essential signals dictating biological outcomes, such as proliferation, adaptation, and differentiation (41). Interestingly, Dolezelova *et al.* recently showed that cytosolic ROS trigger the differentiation of *T. brucei* procyclics into epimastigotes in the *in vitro* differentiation model based on overexpression of RNA-binding protein 6 (RBP6) (42, 43). Indeed, expression of a cytosolic catalase abolished differentiation of the parasite into mature epimastigotes upon induction of RBP6 expression, which was interpreted as the consequence of the degradation of hydrogen peroxide produced by the respiratory chain. Due to its membrane permeability and high stability (44), hydrogen peroxide could be produced in any cell compartment, such as glycosomes, to exert its cytosolic effect. Therefore, FRDg could participate in the production of ROS used to trigger differentiation. This hypothesis is particularly relevant under the glucose-depleted conditions encountered in the midgut of the fly, which resembles the situation faced by the Δ*pepck* mutant grown in rich medium (see Fig. 1), *i.e.*, no contribution of FRDg to succinate production. We therefore propose that, in the glucose-depleted environment of the midgut of the fly, FRDg may contribute to ROS production signaling differentiation of procyclics into epimastigotes. Our model is strengthened by the previous observation that the absence of FRDg (^*RNAi*^FRDg cell lines) does not affect growth of the PCF trypanosome in glucose-rich and glucose-depleted conditions, since the parasite has developed alternative means to maintain the glycosomal redox balance and excrete fumarate instead of succinate from glucose metabolism (14, 20, 21).

## Experimental procedures

### Trypanosomes, cell cultures, and preparation of glycosomal fractions

The PCF of *T. brucei* EATRO1125.T7T (TetR-HYG T7RNAPOL-NEO) was cultivated at 27 °C in the presence of 5% CO_2_ in SDM79 medium containing 10% (v/v) heat-inactivated fetal calf serum and 3.5 mg ml^−1^ hemin (45). Subcellular fractions enriched in glycosomes were prepared by differential centrifugation of parental and Δ*ppdk*/Δ*pepck*/^*RNAi*^GPDH.i PCF trypanosomes as described in (19), after homogenizing the cells with silicon carbide as grinding material. Briefly, 5 × 10^9^ cells were washed once in 50 ml of STE (25 mM Tris, 1 mM EDTA, 250 mM sucrose, pH 7.8). After centrifugation, the pellet was resuspended in 0.5 ml of homogenization buffer STE (STE supplemented with ‘Complete EDTA-Free’ protease-inhibitor cocktail, Roche Applied Science) and ground in a prechilled mortar with 1.5 g of wet-weight silicon carbide per gram of cell pellet. The cells were microscopically checked for at least 90% disruption. The cell lysate was diluted in 7 ml of homogenization buffer, centrifuged at 1000*g* and then at 5000*g* for 10 min each, at 4 °C. The supernatant was centrifuged at 33,000*g* for 10 min at 4 °C to yield the glycosome-enriched pellet, which was resuspended in 2 ml of STE buffer and loaded on a continuous sucrose gradient (1–2 M sucrose in STE). After centrifugation at 39,000 rpm in a vertical rotor, the band corresponding to glycosomes was collected, five times diluted in STE, and centrifuged at 33,000*g* for 30 min at 4 °C to yield the glycosomal pellet, which was resuspended in 0.5 ml of STE.

### Mutant cell lines

The single *Δpepck::BSD/Δpepck::PAC* (Δ*pepck*) and double *Δppdk::TetR-HYG/Δppdk::T7RNAPOL-NEO Δpepck::BSD/Δpepck::PAC* (Δ*ppdk*/Δ*pepck*) null mutant cell lines have been generated before (14, 18, 20, 24). RNAi-mediated inhibition of gene expression of the glycosomal glycerol-3-phosphate dehydrogenase gene (*GPDH*, EC 1.1.1.8, Tb927.8.3530) was performed in the Δ*ppdk*/Δ*pepck* PCF by expression of stem-loop “sense-antisense” RNA molecules of the targeted sequences corresponding to a 564-bp fragment (position 223–786) of the *GPDH* gene (46, 47), using the pLew100 expression vector, which contains the phleomycin resistance gene (kindly provided by E. Wirtz and G. Cross) (48). Similarly, the same approach was used to downregulate expression of the chimeric FRDg-m2 isoform in the Δ*pepck∗* cell line, by targeting a 544-bp fragment (position 1937–2480) of the *FRDm2* gene (Tb927.5.940). The resulting pLew100-GPDH-SAS and pLew100-FRDm2-SAS plasmids containing a sense and antisense version of the targeted gene fragment, separated by a 58-bp and 50-bp fragment, respectively, under the control of a PARP promoter linked to a prokaryotic tetracycline operator, were constructed as previously described using the HindIII, XhoI, and BamHI restriction sites (18, 20). To express the FRDg isoform in the Δ*pepck* background, the *FRDg* gene (Tb927.5.930) was inserted in the HindIII and BamHI restriction sites of the pLew100 vector to produce pLew100-FRDg plasmid. The *FRDg*-Δ*SKI* recombinant gene coding for a FRDg isoform lacking the three C-terminal residues forming the PTS1 (SKI tripeptide) was generated by replacing the 233-bp ApaI/BamHI fragment of the pLew100-FRDg plasmid by the same fragment missing the nine residues coding for the SKI tripeptide. The *FRDg-Δctl* recombinant gene coding for an FRDg isoform lacking the central FRD domain was generated by removing the 1361-bp PvuII/PspOMI fragment of the pLew100-FRDg plasmid, corresponding to position 997 bp and 2358 bp in the *FRDg* gene, followed by recircularization of the resulting plasmid. To produce the *FRDg-ΔNterm* recombinant genes, a 2343-bp PCR fragment corresponding to position 1083 bp to 3426 bp of the *FRDg* gene was inserted into the HindIII and BamHI restriction sites of the pLew100 vector. To generate the *FRDg-ΔNterm/ctl* and *FRDg-Δ2-9* constructs, the 1504-bp HindIII/XhoI fragment of the pLew100-FRDg plasmid, encoding the first 498 amino acids of FRDg, was replaced by a 498-bp HindIII/XhoI fragment and a 1477-bp HindIII/XhoI fragment, respectively.

To constitutively express EGFP in the Δ*pepck∗* cell line, the EGFP sequence was inserted in the HindIII and BamHI restriction sites of the pLew100 vector, which was modified by removing the two tetracycline operator sequences. The pLew100-FRDg-m2-SAS, pLew100-FRDg, and pLew100-EGFPct plasmids designed to generate the Δ*pepck∗*/^*RNAi*^FRDg-m2, Δ*pepck∗*/^*OE*^FRDg, and Δ*pepck∗*/^*OE*^EGFPct cell lines were provided by the Genecust company. The plew100 recombinant plasmids were linearized with the restriction enzyme NotI and transfected into the Δ*ppdk*/Δ*pepck* (pLew100-GPDH-SAS), Δ*pepck∗* (all the other plasmids), or parental (pLew100-FRDg, pLew100-FRDg-ΔSKI and pLew100-FRDg-Δctl) cell lines.

Selection of all these mutant cell lines was performed in SDM79 medium containing hygromycin (25 μg ml^−1^), neomycin (10 μg ml^−1^), blasticidin (10 μg ml^−1^), puromycin (1 μg ml^−1^), and/or phleomycin (5 μg ml^−1^). Aliquots were frozen in liquid nitrogen to provide stocks of each line that had not been in long-term culture. Induction of RNAi cell lines was performed by addition of 1 μg ml^−1^ tetracycline.

### Competitive growth assay

The objective of this assay is to determine slight but significant doubling time difference between a conditional mutant and an EGFP-tagged reference cell line, upon co-culture experiments. This assay is based on the co-culture of a tetracycline-inducible conditional mutant cell line and a reference cell line constitutively expressing EGFP (Δ*pepck∗*/^*OE*^EGFPct_F5), which has a doubling time of 14.26 ± 1.06 h. The SDM79 medium was inoculated with the Δ*pepck∗*/^*OE*^EGFPct_F5 reference cell line (1.4 × 10^6^ cells ml^−1^) and a mutant cell line (0.6 × 10^6^ cells ml^−1^), in the presence or the absence of 1 μg ml^−1^ tetracycline, and the proportion of EGFP-positive cells was determined every day by flow cytometry using a Guava EasyCyte Flow Cytometer (Merck Millipore). The difference of the percentage of EGFP negative cells between induced and noninduced conditions was plotted as a function of time of growth, in order to estimate the growth difference between the noninduced and tetracycline-induced cell line.

### Western blot analyses

Total protein extracts (3–5 × 10^6^ cells) or glycosomal extracts of the parental (EATRO1125.T7T) or mutant PCF of *T. brucei* were separated by SDS-PAGE (8% or 10%) and immunoblotted on TransBlot Turbo Midi-size PVFD Membranes (Bio-Rad) (49). Immunodetection was performed as described (49, 50) using as primary antibodies the rabbit anti-FRD (αFRD, 1:100) (21), the rabbit anti-FRDg (αFRDg, 1:100) (22), the rabbit anti-FRDm2 (αFRDm2, 1:100, produced by Proteogenix from the EISKSVFPDASLGV and ELGHNKSNIVTL peptides), the rabbit anti-PEPCK (αPEPCK, 1:1000) (20), the rabbit anti-GPDH (αGPDH, 1:1000) (51), the rabbit anti-PPDK (αPPDK, 1:1000) (52), the rabbit anti-enolase (αENO 1:100,000, gift from P. Michels, Edinburgh, UK), the rabbit anti-GAPDH (αGAPDH 1:10,000, gift from P. Michels, Edinburgh, UK), the rabbit anti-PFK (αPFK 1:5000, gift from P. Michels, Edinburgh, UK) and the rabbit antibody against the glycosomal isocitrate dehydrogenase, anti-IDHg (αIDHg 1:20,000, produced by Pineda against recombinantly expressed full-length IDHg). Anti-rabbit IgG conjugated to horseradish peroxidase (Bio-Rad, 1:5000 dilution) was used as secondary antibody. Detection was performed using the Clarity Western ECL Substrate as described by the manufacturer (Bio-Rad). Images were acquired and analyzed with the ImageQuant LAS 4000 luminescent image analyzer. For near-infrared fluorescent western blotting, the mouse anti-PFR-A/C (αPFR, 1:2000) (53) and the rabbit anti-FRD (αFRD, 1:1000) (21) were used as primary antibodies, the IR-BLOT 800 anti-mouse IgG (Cyanagen Srl, 1:5000 dilution) and IRDye 680LT anti-rabbit IgG (LI-COR Bioscience, 1:5000 dilution) as secondary antibodies. Image acquisition was performed with the Odyssey CLx Near-Infrared Fluorescence Imaging System and the dedicated software Image Studio (LI-COR Bioscience).

### Analysis of FRDg flavinylation

As described in (54), gels resulting from SDS-PAGE were scanned with a Typhoon TRIO Variable Mode Imager System (GE Healthcare) at λex = 488 nm and λem = 526 nm for detection of covalently bound flavin and at λex = 670 nm and λem = 633 nm for visualization of the Blue Prestained Protein Standard (NEB).

### Cellular ROS measurements

The cellular ROS levels were determined using the 2′,7′-dichlorofluorescein diacetate (H_2_DCFHDA) fluorescent dye. Cells in the exponential growth phase were treated with 10 μM H_2_DCFHDA for 30 min at 27 °C. A total of 10^7^ cells were pelleted (1300*g*, 10 min, RT), washed with 1 ml of PBS (pH 7.4), resuspended in 2 ml of PBS, and immediately analyzed by fluorimetry at 520 nm (Ex: 485 nm) with the FLUOstar Omega (BMG Labtech).

### Digitonin permeabilization

Digitonin permeabilization was performed as described before (18). Briefly, trypanosomes were washed two times in cold PBS and resuspended at 6.5 × 10^8^ cells ml^−1^ (corresponding to 3.3 mg of protein per ml) in STE buffer (250 mM sucrose, 25 mM Tris, pH 7.4, 1 mM EDTA) supplemented with 150 mM NaCl and the Complete Mini EDTA-free protease inhibitor cocktail (Roche Applied Bioscience). Cell aliquots (200 μl) were incubated with increasing quantities of digitonin (Sigma) for 4 min at 25 °C, before centrifugation at 14,000*g* for 2 min to collect the cellular pellet.

### Enzymatic activities

Sonicated (5 s at 4 °C) crude extracts of trypanosomes resuspended in cold hypotonic buffer (10 mM potassium phosphate, pH 7.8) were tested for enzymatic activities. NADH-dependent FRD, glycerol kinase, and malic enzyme activities were measured at 340 nm *via* oxidation of NADH or NADPH, according to published procedures (18).

### Southern blot analysis

Genomic DNA (10 μg) from the parental (EATRO1125.T7T) and Δ*ppdk*/Δ*pepck*/^*RNAi*^GPDH cell lines, extracted as previously described (55), was digested with the NcoI, PvuI, NdeI, or XhoI restriction enzymes, separated by electrophoresis in a 0.8% agarose gel, and transferred onto a nylon membrane (Hybond N^+^, Roche Molecular Biochemicals). The membrane was hybridized with digoxigenin-labeled DNA probes synthesized with a PCR DIG probe synthesis kit (Roche Molecular Biochemicals) as recommended by the supplier. The *FRD* probe was generated by PCR amplification, using the primer pair 5′-GTGTAACGTCGTTGCTCAGTGAGA-3′/5′-GCGAAATTAAATGGGCCCCGCGACG-3′. Probe–target hybrids were visualized by a chemiluminescent assay with the DIG luminescent detection kit (Roche Molecular Biochemicals), according to the manufacturer's instructions. Blots were exposed to ImageQuant LAS4010 (GE Healthcare Life Sciences) for approximately 20 min.

### Label-free quantitative proteomics

Three independent biological replicates for each mutant cell line and control for experiments on total extracts or on glycosome-enriched fractions of trypanosomes have been performed. In total, 10 μg of proteins was loaded on a 10% acrylamide SDS-PAGE gel and proteins were visualized by Colloidal Blue staining. For total extracts, migration was performed classically and each protein lane was cut into four equal segments. For the glycosome-enriched fractions, migration was stopped when samples had just entered the resolving gel and the unresolved region of the gel was cut into only one segment. Finally, each SDS-PAGE band was cut into 1 mm × 1 mm gel pieces. Gel pieces were destained in 25 mM ammonium bicarbonate (NH_4_HCO_3_), 50% Acetonitrile (ACN) and shrunk in ACN for 10 min. After ACN removal, gel pieces were dried at room temperature. Proteins were first reduced in 10 mM dithiothreitol, 100 mM NH_4_HCO_3_ for 30 min at 56 °C, then alkylated in 100 mM iodoacetamide, 100 mM NH_4_HCO_3_ for 30 min at room temperature, and shrunken in ACN for 10 min. After ACN removal, gel pieces were rehydrated with 50 mM NH_4_HCO_3_ for 10 min at room temperature. Before protein digestion, gel pieces were shrunken in ACN for 10 min and dried at room temperature. Proteins were digested by incubating each gel slice with 10 ng μl^−1^ of trypsin (V5111, Promega) in 40 mM NH_4_HCO_3_, 10% ACN, rehydrated at 4 °C for 10 min, and finally incubated overnight at 37 °C. The resulting peptides were extracted from the gel by three steps: a first incubation in 40 mM NH_4_HCO_3_, 10% ACN for 15 min at room temperature and two incubations in 47.5% ACN, 5% formic acid for 15 min at room temperature. The three collected extractions were pooled with the initial digestion supernatant, dried in a SpeedVac, and resuspended with 0.1% formic acid for a final concentration of 0.05 μg μl^−1^. Online nanoLC-MS/MS analyses were performed using an Ultimate 3000 system (Dionex) coupled to a nanospray LTQ Orbitrap XL mass spectrometer (Thermo Fisher Scientific). In total, 0.5 μg of digested protein extracts was loaded on a 300 μm ID × 5 mm PepMap C_18_ precolumn (LC Packings, Dionex, USA) at a flow rate of 10 μl min^−1^. After 5 min desalting, peptides were online separated on a 75 μm ID × 15 cm C_18_PepMap column (LC packings, Dionex, USA) with a 2–40% linear gradient of solvent B (0.1% formic acid in 80% ACN) during 108 min. The separation flow rate was set at 200 nl min^−1^. The mass spectrometer operated in positive ion mode at a 1.8 kV needle voltage and a 42 V capillary voltage. Data were acquired in a data-dependent mode alternating an FTMS scan survey over the range m/z 300–1700 with the resolution set to a value of 60,000 at m/z 400 and six ion trap MS/MS scans with Collision-Induced Dissociation (CID) as activation mode. MS/MS spectra were acquired using a 3 m/z unit ion isolation window and normalized collision energy of 35. Mono-charged ions and unassigned charge-state ions were rejected from fragmentation. Dynamic exclusion duration was set to 30 s. For protein identification, Sequest HT and Mascot 2.4 algorithms through Proteome Discoverer 1.4 Software (Thermo Fisher Scientific Inc) were used for protein identification in batch mode by searching against a *T. brucei* protein database (11,119 entries, release 46). This database was downloaded from http://tritrypdb.org website. Two missed enzyme cleavages were allowed. Mass tolerances in MS and MS/MS were set to 10 ppm and 0.6 Da. Oxidation (M), acetylation (K), and deamidation (N, Q) were searched as dynamic modifications, and carbamidomethylation (C) was searched as static modification. Peptide validation was performed using Percolator algorithm (56), and only “high confidence” peptides were retained corresponding to a 1% False Discovery Rate (FDR) at peptide level. Raw LC-MS/MS data were imported in Progenesis QI (version 2.0; Nonlinear Dynamics, a Waters Company) for feature detection, alignment, and quantification. All sample features were aligned according to retention times by manually inserting up to 50 landmarks followed by automatic alignment to maximally overlay all the two-dimensional (m/z and retention time) feature maps. Singly charged ions and ions with higher charge states than six were excluded from analysis. Univariate one-way analysis of variance (ANOVA) was performed within Progenesis to calculate the protein *p*-value according to the sum of the normalized abundances across all runs. Only proteins with a *p*-value cutoffs <0.05 were validated. A minimum of two unique peptides matched to a protein, and a ≥2-fold change in relative abundance between the two conditions: mutant cell lines *versus* control (three independent biological replicates for each group) were used as the criteria for identification as a differentially expressed protein. Noticeably, only nonconflicting features and unique peptides were considered for calculation at protein level. The mass spectrometry proteomics data have been deposited to the ProteomeXchange Consortium *via* the PRIDE (57) partner repository with the data set identifier PXD020185.

### Analysis of excreted end products from the metabolism of glucose and proline by proton NMR

2 × 10^7^ *T. brucei* PCF was collected by centrifugation at 1400*g* for 10 min, washed once with phosphate-buffered saline (PBS), and incubated in 1 ml (single point analysis) of PBS supplemented with 2 g l^−1^ NaHCO_3_ (pH 7.4). Cells were maintained for 6 h at 27 °C in incubation buffer containing 4 mM [U-^13^C]-glucose and 4 mM nonenriched proline. The integrity of the cells during the incubation was checked by microscopic observation. The supernatant (1 ml) was collected and 50 μl of maleate solution in D_2_O (10 mM) was added as internal reference. H-NMR spectra were performed at 500.19 MHz on a Bruker Avance III 500 HD spectrometer equipped with a 5 mm cryoprobe Prodigy. Measurements were recorded at 25°. Acquisition conditions were as follows: 90° flip angle, 5000 Hz spectral width, 32 K memory size, and 9.3 s total recycle time. Measurements were performed with 64 scans for a total time close to 10 min 30 s. Resonances of the obtained spectra were integrated and metabolites concentrations were calculated using the ERETIC2 NMR quantification Bruker program.

## Data availability

The mass spectrometry proteomics data have been deposited to the ProteomeXchange Consortium (http://proteomecentral.proteomexchange.org) *via* the PRIDE partner repository with the data set identifier PXD020185.

## Supporting information

This article contains supporting information.

## Conflict of interest

The authors declare that they have no conflicts of interest with the contents of this article.
