# Supplementary material for: Metabolic selection of a homologous recombination-mediated gene loss protects Trypanosoma brucei from ROS production by glycosomal fumarate reductase
Source: J Biol Chem. 2021 Mar 17;296:100548. doi: 10.1016/j.jbc.2021.100548 (PMC8065229; doi:10.1016/j.jbc.2021.100548)
Supplement: Supplementary file 1 — Table S1 and Figure S1 [file mmc1.pdf]

Table S1. Excreted end products from metabolism of non-enriched glucose and [U-^{13}C]-proline metabolism by the tetracycline-induced (.i) and non-induced (.ni) Δ*pepck**/^{OE}FRDg-ΔSKI mutant cell lines.

	**Δ*pepck**/ ^{OE}FRDg-ΔSKI.ni** [a]	**Δ*pepck**/ ^{OE}FRDg-ΔSKI.i**
	nmol.h^{-1}.10^{8} cells^{-1} [b]	
n [c]	3	3
Succinate (glucose [d])	nd [e]	nd
Succinate (proline [d])	233 ±12	221 ±16
Acetate (glucose)	494 ±46	480 ±49
Acetate (proline)	576 ±16	572 ±19
Alanine (glucose)	250 ±18	237 ±12
Alanine (proline)	81 ±4	84 ±7
**Total (glucose)**	**600 ±80**	**571 ±76**
**Total (proline)**	**576 ±27**	**572 ±15**

[a] .i: RNAi cell line induced during 7 days by addition of tetracycline; .ni: non-induced RNAi cell line

[b] The amounts of end products excreted from glucose and proline metabolism are expressed as nmoles excreted per hour and per 10^{8} cells

[c] Number of biological replicates

[d] Carbon source metabolized into succinate

[e] nd: not detectable

Figure S1

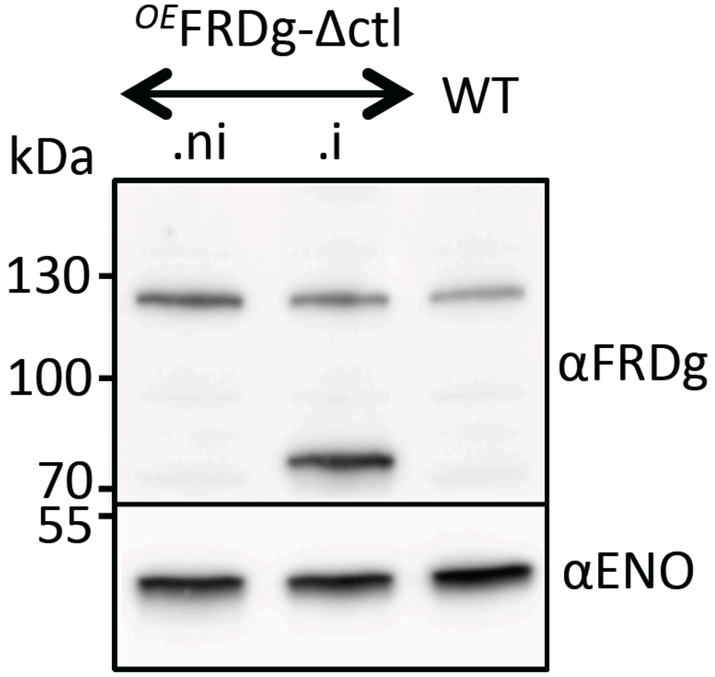

Fig S1. Expression of FRDg-Δctl in the parental (WT) background.
